# Functionalization of Graphite with Oxidative Plasma

**DOI:** 10.3390/ijms23179650

**Published:** 2022-08-25

**Authors:** Paweł Stelmachowski, Dominik Maj, Gabriela Grzybek, Krzysztof Kruczała, Andrzej Kotarba

**Affiliations:** Faculty of Chemistry, Jagiellonian University, Gronostajowa 2, 30-387 Krakow, Poland

**Keywords:** plasma, work function, oxygen functional groups, OFG, graphite, XPS, surface functionalization, carbon materials

## Abstract

Surface-modified graphite is studied as an electrode material, an adsorbent, and a membrane component, among other applications. Modifying the graphite with plasma can be used to create relevant surface functionalities, in particular, various oxygen groups. The application of surface-oxidized graphite often requires its use in an aqueous environment. The application in an aqueous environment is not an issue for acid-oxidized carbons, but a discrepancy in the structure–activity relationship may arise because plasma-oxidized carbons show a time-dependent decrease in the degree of functionalization and related properties. Moreover, plasma-oxidized materials are often characterized in terms of their chemical and physical properties, most notably their degree of functionalization after plasma treatment, without contact with water. In this study, we used low-temperature plasma oxidation with pure oxygen and carbon dioxide and sample-washing with concentrated nitric and sulfuric acids. To evaluate the electronic properties of modified graphite, the work function changes and surface oxygen content were measured just after plasma modification and after water immersion. We show that water immersion drastically decreases the work function of plasma-treated samples, which is accompanied by a decrease in the number of radicals introduced by plasma. Our results demonstrate that the increase in stable work function as a result of plasma treatment, brought about by an increase in the surface oxygen species concentration, can be realized most effectively for the acid-washed graphite.

## 1. Introduction

Natural graphite can be classified into three principal types: crystalline small flake graphite (or flake graphite), crystalline vein or lump graphite, and amorphous graphite (very fine flake graphite). Each natural graphite has different physical properties, appearance, chemical composition, and impurities [1]. Recently, much attention has been focused on the use of economically attractive electrodes based on natural graphite. It is relatively abundant and inexpensive, has good durability and relatively low mass, and has excellent porosity and conductivity [2,3]. Moreover, an improvement in the electrochemical performance of graphite can be achieved through several methods such as mild oxidation, surface deposition of metals/metal oxides, or polymer coating [4]. Such actions may lead to significant changes in the surface of the material, such as the removal of some reactive sites and/or defects, the creation of an oxide layer, the formation of a porous structure, the change in electronic conductivity, and the reduction in the thickness of the solid electrolyte interface layer [5]. To obtain the best properties, the carbon content in graphite must be maximized. It can be achieved by purification with acids such as HCl, HNO_3_, H_2_SO_4_, or HF. According to the authors [6], a maximum carbon content of 98.4% was obtained for acid treatment of flake graphite. The combined acid–alkali–acid (H_2_SO_4_/H_2_O_2_–NaOH–HCl) treatment improved this score to 99.68% or even 99.72% if HF was also employed. Nitric acid treatment is also a very popular method for introducing surface oxygen functional groups (OFG), mainly carboxylic, onto various carbon materials, such as amorphous carbon [7], carbon nanofibers [8], multi- and single-wall carbon nanotubes [9,10], and high surface area graphite [11].

Cold plasma processing is a low-cost, highly effective, and green technology widely used for modification of the surface properties of materials. It is gaining popularity in the fields of biomaterials, adsorbents, polymers, electrodes, and others [12,13,14]. Depending on the application, plasma treatment is used to clean, coat/deposit, or functionalize the surface [14]. The degree of surface changes depends on plasma parameters such as generator power, total gas pressure, and plasma treatment time [13,15]. Optimization of the plasma modification process is extremely important because excessive surface treatment can lead to degradation and destruction of the sample. Moreover, it should be emphasized that the effect of plasma treatment evolves with time after plasma removal because of the variability of the state of the plasma-generated surface functional groups. Their orientation changes towards lowering the surface energy; they may diffuse on the surface or recombine and eventually leave the surface [15]. When plasma-modified material is applied in an aqueous environment, special attention should be paid to the characteristics after immersion.

The degree of plasma modification of the material surface can be followed by the measurements of changes in the work function (WF). The work function of a material is defined as the minimum energy required to move an electron from the Fermi level into the vacuum. Its value provides information on the electronic properties of the solid surface [16]. Since the work function is directly related to the electron donor properties, its studies can be successfully used to optimize the surface state of many materials. To this end, Duch et al. presented a substantial increase of about 1 eV in the work function of the graphenic sheets upon oxygen plasma treatment. The authors also revealed that the plasma-modified electronic properties change toward the initial state with time; however, the final value of the work function after 2 months was still higher than for the native sample [15]. The literature survey indicates the particular significance of plasma treatment in the area of carbon materials. Their properties are tailored by plasma modification in many different applications [13,14,15,17,18]. In [19], the authors described the positive influence of plasma treatment (plasma N_2_ + H_2_) on the graphite anode in lithium ion batteries. The authors were working with graphite powders with a mean particle size of about 3 μm. The observed improvement in electrochemical performance (exceeding the theoretical limit of the capacity) resulted from significant structural modifications of the surface of graphite, including the introduction of N atoms into the graphite lattice sites. High-surface-area graphites, used as catalyst support in a variety of chemical reactions, are often modified with plasma to create a surface with a high concentration of catalytic active sites [17]. The oxygen plasma treatment is a dry and environmentally friendly alternative to conventional chemical and electrochemical oxidation in gaseous (O_2_, O_3_, CO_2_) or liquid media (acids, H_2_O_2_). In turn, carbon biomaterials are treated with plasma to ensure their resistance to bacterial adhesion. The attachment of pathogenic bacteria that possess a net negative charge to biomaterials has been reported to be strongly correlated with their work function [18,20]. Epifanio et al. demonstrated a positive effect of air plasma treatment on the electrochemical adhesion of active microorganisms to the graphite electrode [21]. Atmospheric air plasma treatment of the electrode surface introduced hydrophilic functional groups, leading to an increase in cell adhesion and electroactivity. Such results indicate that air plasma pretreatment is an effective option for increasing the output current in bioelectrochemical systems. The application of plasma for the functionalization of carbon-based materials for electrochemistry is noteworthy. In [22], the authors used a dual oxygen plasma oxidation approach followed by H_2_O_2_ treatment to functionalize graphite felt electrodes for an all-vanadium flow-through battery system. This combination of treatments increased the energy efficiency of the cell by 8.2% compared to oxidation by thermal treatment in air. The use of the above-mentioned solution may reduce the cost of vanadium flow-through battery cells, and thus increase the scale of their application. The reactivity of carbon-based electrocatalysts, for example, for the oxygen reduction reaction, is correlated with the functional oxygen groups present in the carbon materials [23] and can be successfully improved by using plasma. A clear correlation between their electrocatalytic activity and work function indicates that the latter can be a common descriptor of activity [24]. We recently reported that the degree of functionalization of the plasma-modified graphene paper is controlled by the post-plasmatic reactions in water. Immersion in water of plasma-treated graphene paper resulted in stable surface functionalization, confirmed by the constant value of the work function [25].

The need for preliminary purification of natural graphite before its application and possible surface functionalization with oxygen groups motivated us to investigate the effect of acid washing on plasma oxidation. We aimed to evaluate the effect of plasma treatment on the electronic properties and surface composition of the off-the-shelf graphite material washed with different acids. We used oxygen and carbon dioxide plasmas to evaluate the extent of possible WF changes and confronted the results for two approaches: plasma oxidation of the off-the-shelf sample and the same treatment of graphite, which underwent acid purification.

## 2. Results

**Figure 1:**
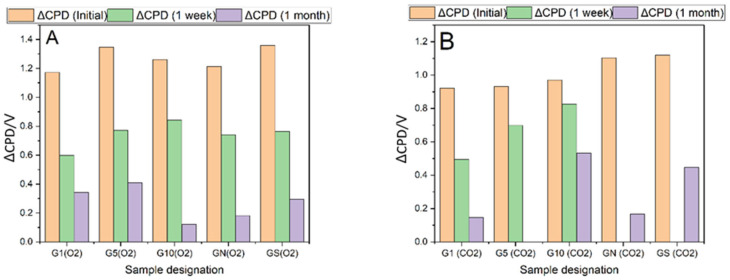
Work function changes after one week and one month after plasma treatment of of-the-shelf and acid-treated graphite samples. (**A**) oxygen plasma-G(O2), (**B**) CO_2_ plasma-G(CO2). GN-nitric acid-washed graphite, GN(O2) and GN(CO2)-plasma-treated nitric acid-washed graphite, GS–sulfuric acid-washed graphite, GS(O2) and GS(CO2) plasma-treated sulfuric acid-washed graphite.

The most spectacular changes are observed by following the contact potential difference (CPD) with the Kelvin probe for the samples before and after plasma treatment, Figure 1. The initial increase in the CPD is of the order of 1 V, equivalent to an increase of 1 eV of the work function of the material. The observed modification of the electronic properties of the surface is not stable over time and decreases substantially, with the magnitude of the effect depending on the type of plasma (O_2_, CO_2_) and the time of treatment. As presented in Figure 1 CO_2_ plasma treatment results in a higher decrease in CPD than O_2_ plasma. The graphs of the short-term evolution of CPD for different plasma treatments are presented in Figures S1 and S2 in Supplementary Materials. Acid-washed samples exhibit a similar pattern of high initial increase in CPD followed by substantial decrease over time.

**Figure 2:**
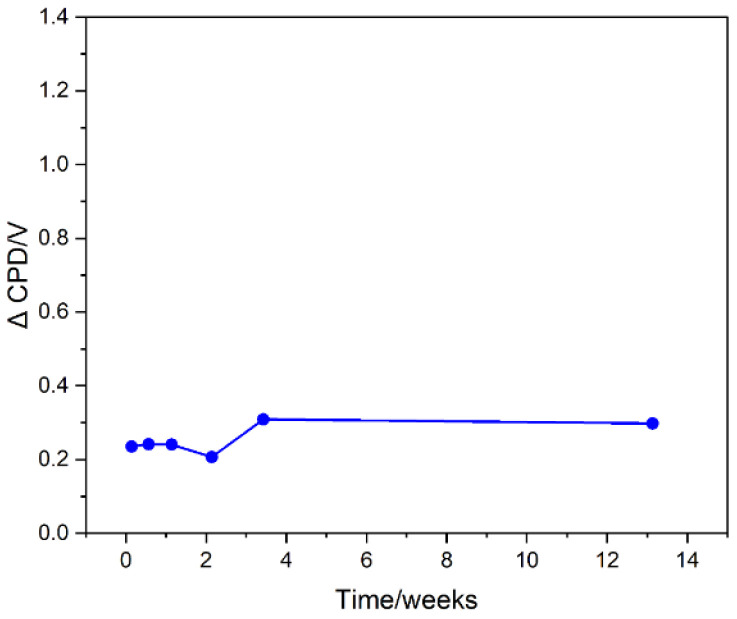
Contact potential difference of 10 min oxygen plasma-treated graphite immersed in water.

To obtain a stable surface of the plasma-modified graphites, the samples were washed with deionized water. Such a treatment was chosen to remove the static electricity charging of the samples created by plasma treatment and to promote surface reactions that lead to changes in surface composition. This treatment allowed the material to have stable surface properties, as evidenced by a constant value of the change in work function (change in CPD) relative to the starting material (Figure 2).

**Figure 3:**
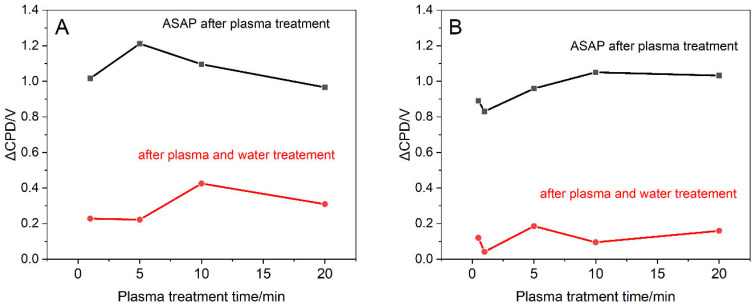
Changes in graphite surface contact potential difference (work function) for different times of plasma treatment with O_2_ (**A**) and CO_2_ (**B**). ASAP–CPD measurement performed as soon as possible after plasma treatment.

The effect of water for different plasma treatment times for O_2_ and CO_2_ gases is presented in Figure 3. An immediate decrease in work function is easily observed, and the final CPD values vary depending on the type of plasma and the treatment time. The increase in final work function due to plasma and water treatment is more pronounced for O_2_ plasma than for CO_2_ plasma. In the case of oxygen plasma, the maximum changes of surface electronic properties (ΔCPD) are observed for 10 min of plasma treatment, while for carbon dioxide plasma the maximum is much less pronounced and is located at 5 min of plasma treatment.

**Figure 4:**
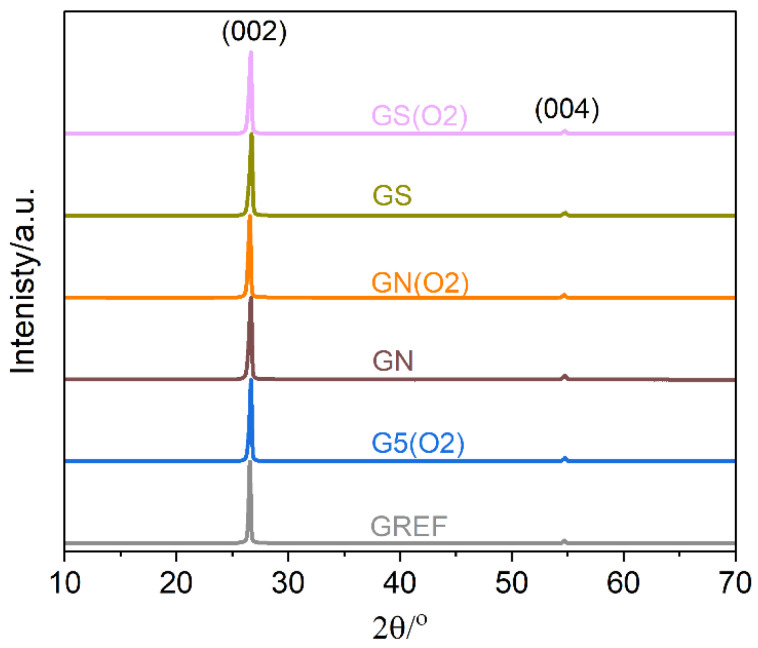
Powder X-ray diffractograms of the studied samples: reference-GREF, 5 min oxygen plasma-treated-G(O2), nitric acid-treated-GN, nitric acid + oxygen plasma-GN(O2) treated, sulfuric acid-treated-GS, and sulfuric acid + oxygen plasma-GS(O2).

XRD characterization was performed to verify the structural integrity of the modified graphite samples; Figure 4. The diffraction patterns exhibit typical two maxima assigned to the crystal planes (002) and (004). Notably, no other phases are detected. Neither plasma nor acid treatments induce noticeable structural changes. To further analyze the data, the crystallite sizes were determined with the Scherrer formula using (002) diffraction maximum [26]. For the unmodified graphite sample, it is equal to about 80 nm, whereas plasma modification results in a slight decrease to 65 nm. Both acid washing treatments result in a decrease in the average crystallite size of graphite to 50 nm, which is not further influenced by plasma oxidation.

**Figure 5:**
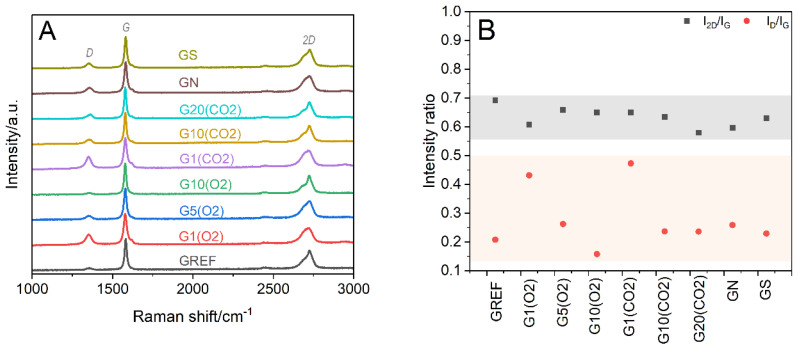
(**A**) Raman spectra of reference (GREF) and oxidized samples: treated with O_2_ plasma for 1 min—G1(O2), 5 min—G5(O2), 10 min—G10(O2); treated with CO_2_ plasma for 1 min—G1(CO2), 10 min—G10(CO2), 20 min—G20(CO2); treated with nitric acid (GN), and treated with sulfuric acid (GS). (**B**) Analysis of I_D_/I_G_ and I_2D_/I_G_ intensity ratios from Raman spectra of studied samples.

The structural characterization by XRD was supported by Raman spectroscopy studies. The Raman spectra of the reference (GREF) and modified graphite samples, treated with oxygen (G(O2)) or carbon dioxide (G(CO2)) plasmas using 1, 5, 10, and 20 min of exposure time, and with nitric or sulfuric acid are presented in Figure 5A. In all cases, intense bands are present at ~1580 (G) and ~2725 cm^−1^ (2D), which are distinctive for graphite. The G band is related to the vibrational mode of graphitic carbon, whereas the 2D band, with typical graphite asymmetry, is the result of the two-phonon lattice vibrational process [27]. In the spectra of the modified samples, the disorder-induced D band at ~1355 cm^−1^ appears with its intensity slightly decreasing with plasma treatment time, while the band associated with the carbon amorphization process at ~1620 cm^−1^ (D’) is almost unnoticed. To assess the degree of disorder of the graphite structure, the I_D_/I_G_ and I_2D_/I_G_ ratios were calculated (Figure 5B). The low intensity of the D band, together with the irrelevant variations in the I_2D_/I_G_ and I_D_/I_G_ values, indicates that the extent of disorder of the bulk graphite structure is negligible, regardless of the modification procedure (plasma versus acid treatment, plasma exposure time). This assertion, reinforced by the highly ordered structure inferred from the XRD results (Figure 3), is in line with previous research [17]. The slight decrease in the I_D_/I_G_ ratio within the G(O2) and G(CO2) series can be attributed to the removal of disordered graphitic layers with a longer plasma exposure time [17]. Similarly, the specific surface area is not influenced by 1 min of oxygen plasma (the same 12 m^2^·g^−1^ before and after) and only slightly decreases after prolonged plasma treatment (8 m^2^·g^−1^ after 20 min). The morphology of the oxidized graphite samples did not differ appreciably from that of the starting material even after a harsh sulfuric acid treatment (scanning electron microscopy, SEM, pictures in Figure S3).

**Figure 6:**
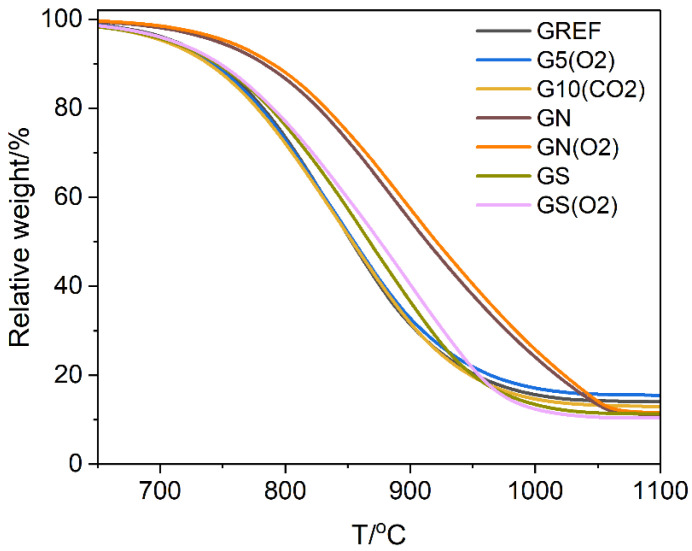
Thermal stability of acid and plasma-treated materials. Reference graphite (GREF) and plasma oxidized samples: treated with O_2_ plasma for 5 min-G5(O2), treated with CO_2_ plasma for 10 min G10(CO2), treated with nitric acid (GN), and treated with sulfuric acid (GS), GS(O2) and GN(O2)-acid washed samples treated with O_2_ plasma for 10 min.

A complementary characterisation of the materials was performed by thermogravimetric studies, Figure 6. Plasma treatment was found to have no influence on the thermal stability of graphite. However, treatment with acids, especially nitric acid, increases thermal stability. The effect is due to the removal of iron oxide impurity, which acts as an oxidation catalyst [28]. The oxygen plasma treatment of the acid-washed samples did not change their thermal stability appreciably, similarly to that of the unwashed graphite.

**Figure 7:**
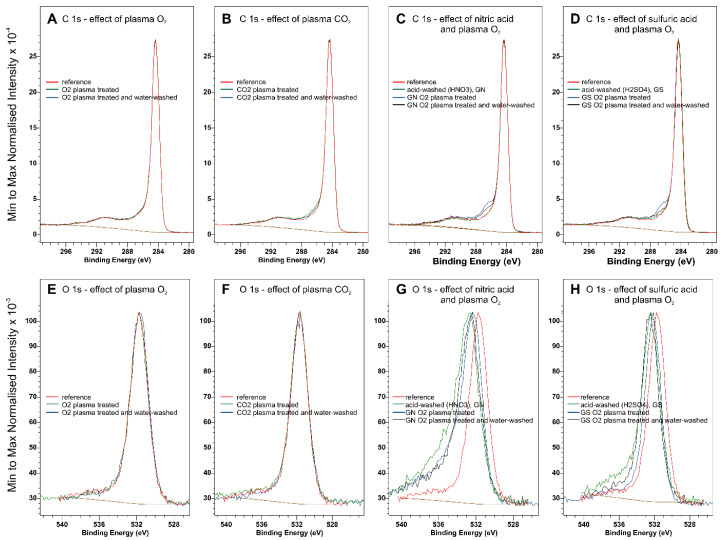
XPS core C 1 s and O 1 s spectra of graphite treated with O_2_ plasma (**A**,**E**), CO_2_ plasma (**B**,**F**), nitric acid (**C**,**G**), and sulfuric acid (**D**,**H**).

Normalized XPS C 1 s and O 1 s spectra of plasma and acid-treated graphites are collected in Figure 7. The full sets of narrow scan XPS spectra for the investigated samples can be found in Supplementary Materials, Figures S4–S14. A comparison of the normalized spectra for O_2_ and CO_2_ plasma modified samples indicates that speciation of the oxygen functional groups does not change. Although the C 1 s spectra of acid-washed graphites (nitric acid-washed, GN, sulfuric acid-washed, GS) do not differ noticeably, plasma treatment in these cases results in an increased intensity of around 286.1 eV, characteristic for C-O-type functional groups [29]. The O 1 s spectra of acid-washed samples differ from those of the starting material, probably due to the removal of inorganic impurities, such as Fe_2_O_3_. Following Table S1, it can be concluded that acid washing removes iron oxide impurities and uncovers SiO_2_, changing the oxygen balance in the sample. To account for the changes in the chemical composition of graphite due to the removal of Fe_2_O_3_ and the uncovering of SiO_2_, the corrected oxygen atomic composition bound in the functional carbon-oxygen groups was obtained by subtracting the oxygen from Fe_2_O_3_ (Fe at.% × 1.5), SiO_2_ (Si at.% × 2), and SO_3_ where adequate (S at.% × 3). The summary of surface quantification with XPS is collected in Table S1, Supplementary Materials.

**Figure 8:**
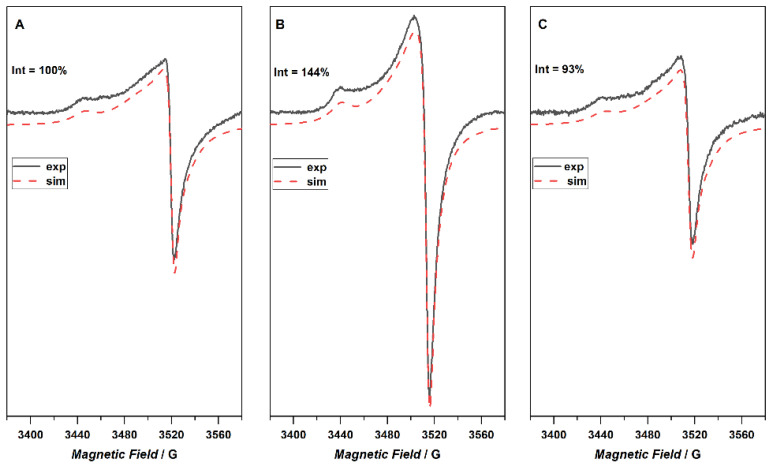
EPR spectra of plasma-treated graphite. (**A**) reference graphite; (**B**) measured as soon as possible after plasma treatment; (**C**) plasma and water treated sample.

The EPR spectra of the investigated samples registered at room temperature are shown in Figure 8. The composite EPR spectrum for the reference sample (GREF) was simulated as a superposition of two components characterized by g_∥_ = 2.0459 and g_⏊_ = 2.0028 for the first one (GREFC-1), while the second center was characterized by g_∥_ = 2.0198 and g_⏊_ = 2.0002 (GREFC-2). The simulation represented a very good fit; however, a small discrepancy can be observed around g~2 which might be attributed to an additional sharp signal. Due to the very small intensity, this line was not included in the simulation. The signal denoted GREFC-1 was attributed to electrons that can travel through a large number of crystallites before a change in spin orientation takes place [30]. The observed shape of the EPR spectrum is due to a time averaging of the anisotropy owing to electron motion. The second signal, GREFC-2, originates from the electron whose spin flipped after only crossing a few crystallites. The signal from plasma treated graphite shows a similar shape and also consists of two components (g_∥_ = 2.0459 g_⏊_ = 2.0027 and SG-2 g_∥_ = 2.0199 g_⏊_ = 2.0039), though, its intensity increased significantly. After immersion of the sample in water, the shape and intensity of the EPR signal returned to that observed for the native graphite (g_∥_ = 2.0459 g_⏊_= 2.0028, and g_∥_ = 2.0198 g_⏊_ = 2.0002).

## 3. Discussion

The physicochemical characterization of modified graphites shows that the applied treatments influence neither the structure nor the morphology of the commercial graphite used. This is expected for plasma treatment, since oxidation is restricted to the near-surface region (Tables S1–S3). The increase in the Si content of SiO_2_ in the plasma-treated samples is a sign of total oxidation of some of the carbon components that reveal the silica impurity. Nevertheless, plasma treatment does not influence the thermal stability of graphite, most likely due to a negligible effect on the structure of the material and the introduction only of unstable oxygen species. In contrast, acid washing increases the thermal stability of the graphite material as a result of the removal of iron impurity, which acts as an oxidation catalyst.

**Figure 9:**
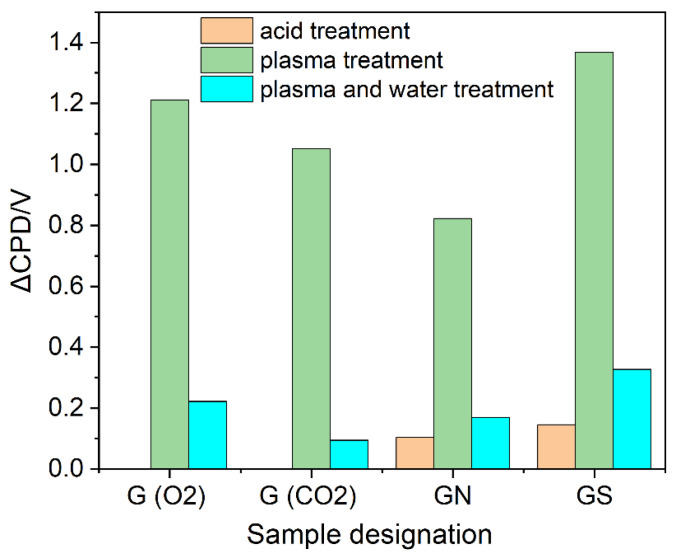
Changes in contact potential difference (changes in work function) of graphite samples—a comparison of acid and plasma treatments. G(O2)—oxygen plasma-treated sample, G(CO2)—CO_2_ plasma-treated sample, GN, GS—nitric acid and sulfuric acid-washed samples later treated with oxygen plasma.

The surface changes of graphite result in an increase in work function due to the mild oxidation. However, the changes are unstable over time and a gradual decrease in the work function is observed. This effect can be explained by considering two components of the modifications: electrostatic and chemical. The former is related to the effect of graphite powder charging with static electricity during plasma treatment and a discharge during measurement with the Kelvin probe, where the sample is grounded. The latter component is responsible for more stable surface modification–the introduction of oxygen functional groups. To equilibrate the samples and remove the charging, graphite powders were immersed in water after plasma treatment. This procedure also decreases the number of unpaired electrons as shown by EPR measurements. The additional effect of such treatment is related to the surface reactivity upon activation in plasma. As expected, for each plasma treatment, the values of the work function (CPD) decreased substantially but were higher than for the original samples (ΔCPD > 0, Figure 9).

**Figure 10:**
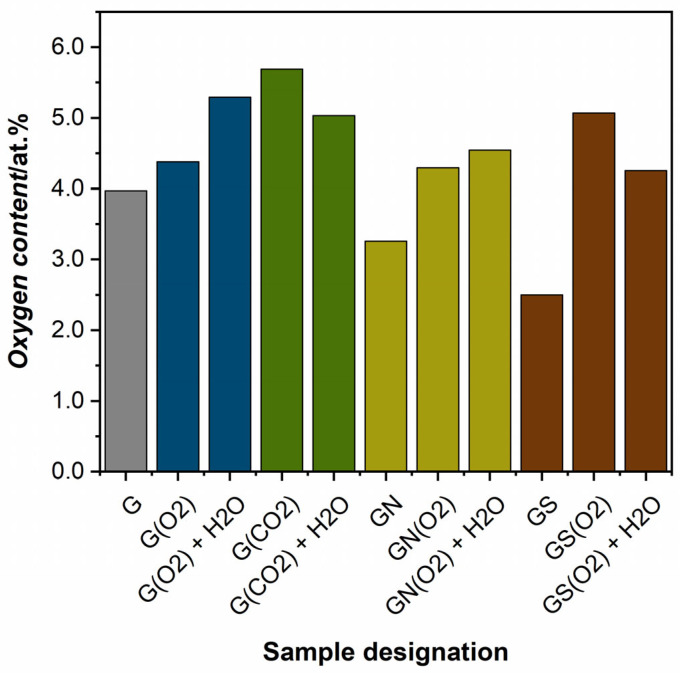
Oxygen content determined with XPS analysis of plasma and acid-treated graphite samples. Legend: G—graphite reference, G(O2)—O_2_ plasma-treated graphite; G(O2) + H_2_O—water-washed G(O2), G(CO2)—CO_2_ plasma-treated graphite, G(CO2) + H_2_O—water-washed G(CO2), GN —nitric acid-washed graphite, GN(O2)—O_2_ plasma-treated graphite GN, GN(O2) + H_2_O—water-washed GN(O2), GS—sulfuric acid-washed graphite, GS(O2)—O_2_ plasma-treated graphite GS, GS(O2) + H_2_O—water-washed GS(O2).

Quantification of oxygen functional groups in graphite samples is shown in Figure 10. The oxygen content (based on Table S1) in the O_2_ and CO_2_ plasma and water-treated samples is higher than in the original graphite, which explains the increase in work function. Interestingly, CO_2_ plasma is more effective in introducing OFGs, but water treatment leads to their decrease. In contrast, oxygen plasma appears to also activate the materials, similarly to the effect reported for graphene paper [25].

The investigated, off-the-shelf graphite contained impurities in the form of SiO_2_ and Fe_2_O_3_. To purify the material, nitric and sulfuric acids were used. Acid treatment resulted in substantial removal of iron oxide impurity. At the same time, the work function of the graphite increased, despite the substantial decrease in the content of the oxygen functional groups. However, as evidenced in Figure 7G,H, the shift in the O 1s spectra may indicate a change in OFG speciation, leading to the modification of the work function. In the case of the sulfuric acid-washed sample, sulfate groups are present on the surface (Figures S12 and S13) possibly adding to the observed increase in work function. However, these groups disappear after oxygen plasma treatment followed by water washing (Figure S14). Moreover, intimate interaction between the graphite and iron impurities may result in a decreased work function of the of-the-shelf graphite. Nevertheless, plasma treatment of the acid-washed samples resulted in the incorporation of stable oxygen functional groups, which further increased the work function graphite.

Plasma, consisting of radicals and free electrons, can transfer these species and charges to the materials in contact. The studied graphite already contains radicals in the form of free electrons, but plasma treatment increases their concentration, as shown in Figure 8. Following the adopted experimental procedure, immersion in water results in the removal of these additional radicals. Electron paramagnetic resonance studies allow for some insight into the state of the graphite samples upon plasma treatment. The increase in the radical signal coincides with the increase in the work function and the surface oxygen. Similarly, the decrease in the radical signal for the water-treated graphite follows the decrease in work function. Changes in the surface oxygen content do not increase uniformly after the washing of the plasma-oxidized samples. Therefore, the reactivity in water—increase or decrease in the number of functional oxygen groups—will depend on the interplay between surface disordering due to ion bombardment, the formation of radicals, as well as the number and type of OFG already present.

## 4. Materials and Methods

Graphite powder (Polskie Odczynniki Chemiczne, POCH) was modified in this work using both acid immersion and plasma treatment. The wet acid modification was carried out by placing 2 g of graphite in a round bottom flask and adding 80 mL of concentrated acid. Sulfuric acid (VI) (Chempur) with a concentration of 95% and nitric acid (V) (POCH Basic) with a concentration of 65% were applied. The mixtures were then heated to a temperature of about 96 °C for 16 h. The product was then filtered on a Buchner funnel and washed with deionized water until pH was close to neutral. The obtained powders were dried and ground in a mortar.

Plasma treatment was carried out using the commercial cold plasma system (Femto-Diener Electronic GmbH, Nagold, Germany) with a generator frequency of 40 kHz, employing pure oxygen (Air Products, 99.9998% O_2_) or CO_2_ (Air Products, X50S 37.5 K, ultra-pure) as feed gases for plasma generation. The optimization of the plasma modification parameters was performed with the use of 100% power and 0.2 mbar pressure for different plasma treatment times: for oxygen plasma, the times were: 1, 5, 10, and 20 min (samples designation: G1(O2), G5(O2), G10(O2), G20(O2)), and for CO_2_ plasma, 1, 5, 10, and 20 min (samples designation: G1(CO2), G5(CO2), G10(CO2), G20(CO2)). The plasma treatment was applied to unmodified graphite and samples already treated with concentrated acids. After plasma treatment, the samples were immersed in deionized water (DI) for surface stabilization and dried at 60 °C. Based on the relative changes in work function before and after water immersion, 5 min of plasma treatment was selected to modify acid-treated samples, as well as samples for electron paramagnetic resonance (EPR) and X-ray photoelectron spectroscopy (XPS) studies.

To determine the effect of applied modifications on the electronic properties of graphite, the work function changes were followed. Work function studies for plasma treatment were performed in a series of three measurements: untreated sample, sample just after plasma treatment, and sample immersed in DI water just after plasma treatment.

Changes in the work function of graphite samples were investigated by measuring contact potential difference (ΔCPD) measurements. The experiments were carried out using the Kelvin probe method with a KP6500 device (McAllister Technical Services, Coeur d’Alene, ID, USA). The stainless steel plate (d = 3 mm) was used as an electrode (WF_ref_ = 4.3 eV). The measurements were carried out under ambient conditions (room temperature, atmospheric pressure), with vibration frequency at 114 Hz and amplitude at 40 a.u.

The structural analysis of the reference and functionalized samples was carried out using a Rigaku Multiflex diffractometer, using Cu Ka radiation (40 kV, 40 mA). Powder X-ray diffractograms were collected in the 2theta range of 10–70° (step size of 0.02° and accumulation time of 3 s). μRaman spectra of the samples were collected using a Renishaw InVia spectrometer equipped with a 514 nm laser. Measurements were carried out in the spectral range of 1000–3000 cm^−1^ with a resolution of 1 cm^−1^. The accumulation of ten scans was applied for each spectrum.

Thermogravimetric analysis of reference and functionalized samples was performed using TGA/DSC 1 equipment (Mettler Toledo). Approximately 10 mg of the sample were heated in the flow of synthetic air 40 mL·min^−1^ (mixed with Ar 20 mL·min^−1^) in the temperature range of 25–1100 °C with a heating rate of 20 degrees per minute.

The surface composition was examined using XPS with a SESR4000 analyzer (Gammadata Scienta, Uppsala, Sweden, the XPS setup was provided by Prevac, Rogów, Poland). The base pressure in a vacuum chamber was below 5 × 10^−9^ mbar, and monochromatized Al-Kα source with the 250 W at 1486.6 eV emission energy was used with the pass energy for selected narrow-range binding energy scans of 100 eV. To process the raw data, CasaXPS Version 2.3.24PR1.0 (Casa Software Ltd., Teignmouth, UK) was used [31].

Local structural changes of modified graphites were investigated using a Renishaw InVia spectrometer equipped with a 514 nm laser. μRaman spectra were collected in the range of 1000–3000 cm^−1^ with a resolution of 1 cm^−1^, by accumulating ten scans for each spectrum.

The number and type of paramagnetic species in the graphite samples were investigated using EPR spectroscopy. Continuous wave EPR spectra were measured with a Bruker Elexsys E500 X-band spectrometer equipped with the Xepr data system for spectra acquisition and manipulation and the super high-sensitivity cavity ER 4122 SHQE. Spectra were recorded with a 100 kHz magnetic field modulation. In standard experiments, the microwave power was 2 mW, sweep width 20 mT, modulation amplitude 0.2 mT, number of points 1024, and 4 scans were acquired. The EPR spectra were simulated using the EPRsim32 program written in Microsoft Visual C^++^ 6.0 using the Microsoft Foundation Class library [32].

## 5. Conclusions

Graphite is being investigated for potential and improved applications in many areas where tuning the surface properties is paramount to its performance. In this study, we evaluated the tuning of the surface electronic properties and surface oxygen content by oxidative plasma modification of the sample of commercial graphite. Since the surface of the plasma-oxidized graphite is unstable over time, simple washing with water was chosen to obtain a stable material. Additionally, because the various graphite samples may differ according to their origin, the effect of acid washing was also evaluated. The main finding of this research is that the combination of acid and plasma treatment is the most effective way to modify the electronic properties and surface functionalization of microcrystalline graphite material.

The nitric and sulfuric acid washing results in a decrease in iron oxide impurity, which increases the thermal stability of graphite. For the investigated graphite samples, the increase in the stable work function due to plasma treatment is caused by (1) an increase in the concentration of surface oxygen species and (2) a change in the speciation of the surface oxygen species. Stable plasma functionalization with oxygen functional groups is most evident in nitric and sulfuric acid washed samples, which is reflected in the highest observed increase in work function for acid-treated samples and a visible increase in the relative content of C-O functional groups.

## Supplementary Materials

The following supporting information can be downloaded at: https://www.mdpi.com/article/10.3390/ijms23179650/s1.
