# Supplementary material for: Functionalization of Graphite with Oxidative Plasma
Source: Int J Mol Sci. 2022 Aug 25;23(17):9650. doi: 10.3390/ijms23179650 (PMC9456250; doi:10.3390/ijms23179650)
Supplement: Supplementary file 1 [file ijms-23-09650-s001.zip › ijms-1840674-supplementary.pdf]

Additional experimental details

Porosimetry measurements were performed using a 3Flex v.1.00 (Micromeritics) automated gas adsorption system with N_2-sorption at −196 °C. The samples were outgassed under a vacuum at 350°C for 24 h before the analysis. The specific surface area (SSA) of the samples was determined using the BET model.

The elemental composition of the graphite samples was examined using X-ray fluorescence spectroscopy (XRF) with ARL Quant'X, ThermoFisher (4–50 kV, 1 kV step, Rh anode, 3.5 mm Si(Li) drifted crystal with a Peltier cooling (~185 K) detector). The quantitative analysis of metallic and other heavy-atom impurities was done with UniQuant software after calibration with a series of metallic standards.

The scanning electron microscopy (SEM) study was carried out for reference and modified graphite with concentrated sulfuric acid (VI). The imaging was performed using a Tescan VEGA 3 scanning electron microscope. The aperture angle was selected individually for each sample. In the first stage, during the measurement, various parameters were changed and the SEM images were interpreted on their basis. During the measurement, the following parameters were changed: accelerating voltage, working distance, and magnification.

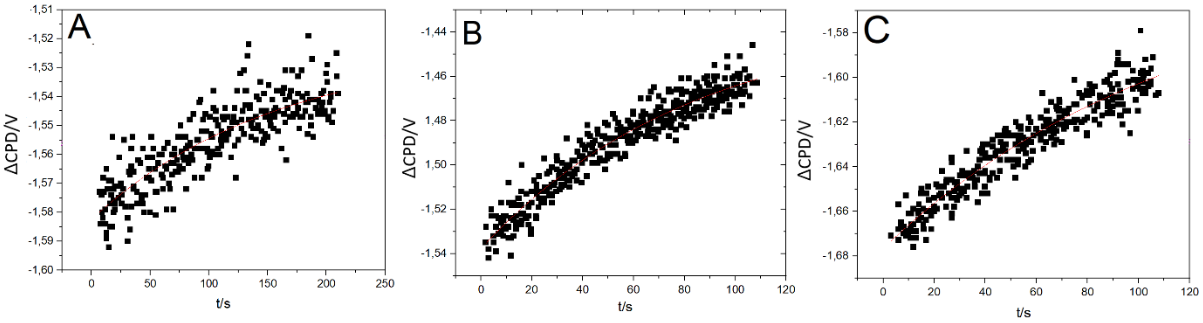

Figure S1. Changes of the measured CPD just after treatment with plasma O_2, A – 1 min, B – 5 min, C – 20 min treatment time.

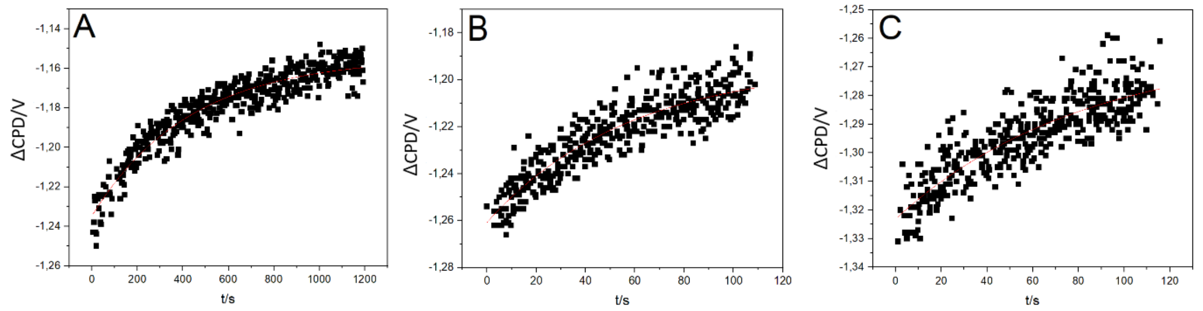

Figure S2. P Changes of the measured CPD just after treatment with plasma CO_2, A – 1 min, B – 5 min, C – 10 min treatment time.

Table S1. Surface chemical composition from XPS. N – nitric acid-treated, S – sulfuric acid-treated, PL – plasma treatment, O_2, CO_2 – plasma gas, H_2O – water treated samples.

	Composition/at.%					
**Sample Identifier**	**Si 2p**	**Fe 2p**	**S 2p**	**C 1s**	**O 1s**	**O***
reference graphite (G)	3.3	1.3	0.4	81.0	13.9	4.0
G N	4.6	0.2	0.0	82.3	12.9	3.3
G N PLO_2	5.7	0.3	0.0	78.0	16.1	4.3
G N O2, H_2O	4.2	0.1	0.0	82.5	13.2	4.5
G S	4.5	0.1	0.2	82.9	12.3	2.5
G S PLO_2	5.2	0.2	0.3	77.6	16.7	5.1
G S O_2, H_2O	4.5	0.3	0.1	81.0	14.1	4.3
G PLO_2	3.6	1.4	0.5	79.3	15.2	4.4
G PLO_2 H_2O	3.6	1.7	0.1	79.4	15.2	5.3
G PLO_2 H_2O, repeated	4.0	1.4	0.1	78.5	15.9	5.4
G PLCO_2	3.8	1.3	0.3	78.3	16.3	5.7
G PLCO_2 H_2O	3.7	1.6	0.0	79.8	14.9	5.0

* corrected by subtracting 2xSi at.% (SiO_2), 1.5xFe at.% (Fe_2O_3), 3xS at.% (SO_3).

Table S2. Chemical composition of studied graphites derived from XRF.

	Composition/wt.%		
**Sample Identifier**	**Si**	**Fe**	**K**
Reference graphite (G)	0.792	0.687	0.079
G PLO_2 5min	0.929	0.315	0.072
G PLCO_2 10min	0.984	0.341	0.083
G S	1.350	0.089	0.092
G N	1.550	0.036	0.099

Table S3. Chemical composition from the elementary analysis.

	Composition/wt.%		
**Sample Identifier**	**N**	**C**	**H**
Reference graphite (G)	0.05	89.09	0.259
G S	0.08	92.40	0.272
G N	0.08	93.29	0.327
GS PLO_2 5 min H_2O	0.12	91.87	0.345
GN PLO_2 5 min H_2O	0.11	92.87	0.376
G PLO_2 5 min H_2O	0.12	89.58	0.343
G PLCO_2 10 min H_2O	0.11	87.50	0.332

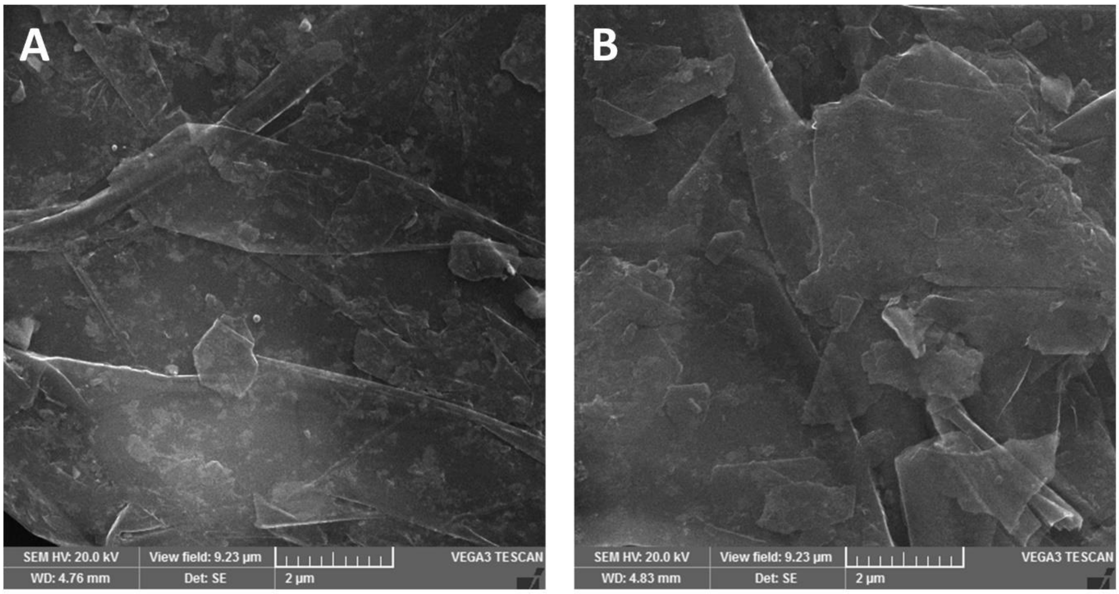

Figure S3. SEM pictures of A) reference graphite and B) sulfuric acid-washed graphite.

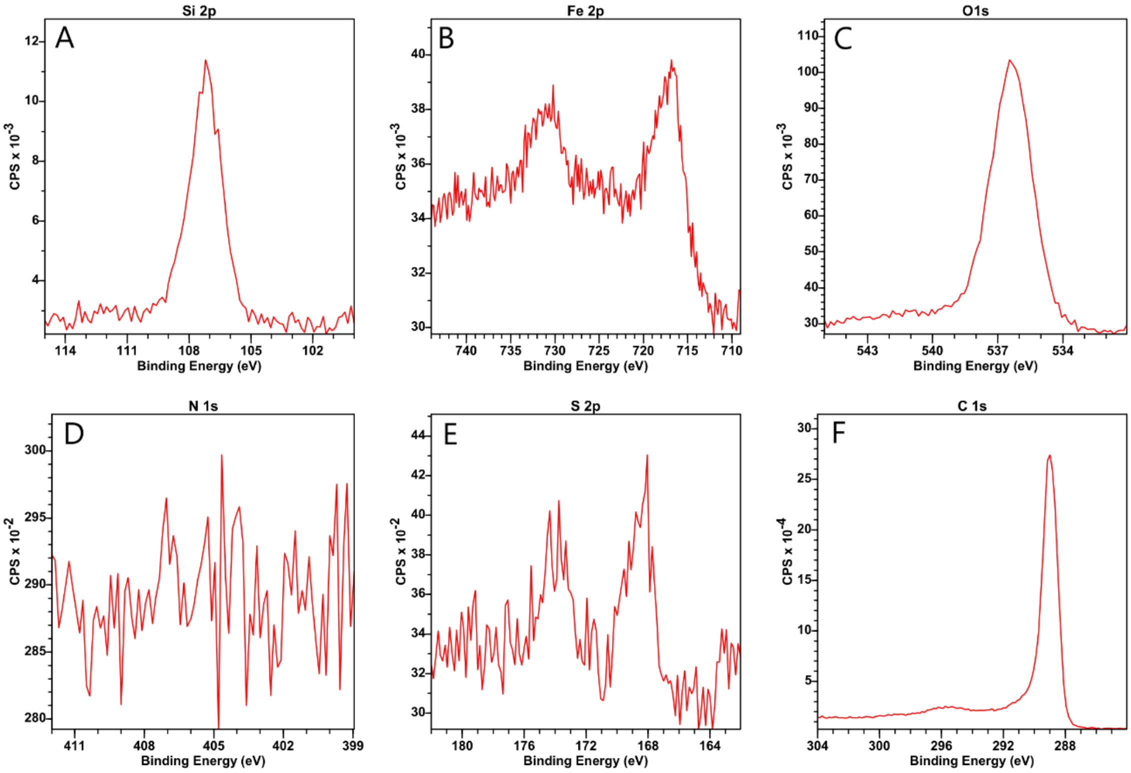

Figure S4. Narrow scan XPS spectra of **reference** graphite. A) Si 2p, B) Fe 2p, C) O 1s, D) N 1s, E) S 2p, F) C 1s.

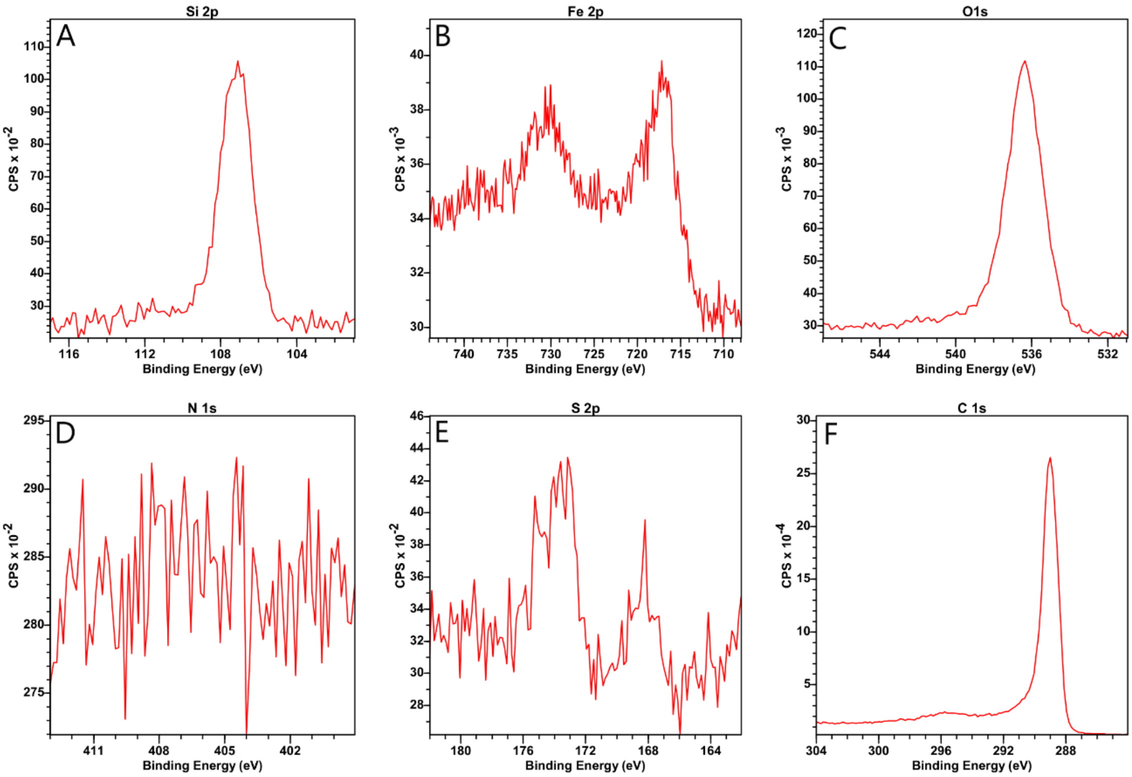

Figure S5. Narrow scan XPS spectra of graphite treated with **plasma O_2**, 10 min – just after plasma treatment. A) Si 2p, B) Fe 2p, C) O 1s, D) N 1s, E) S 2p, F) C 1s.

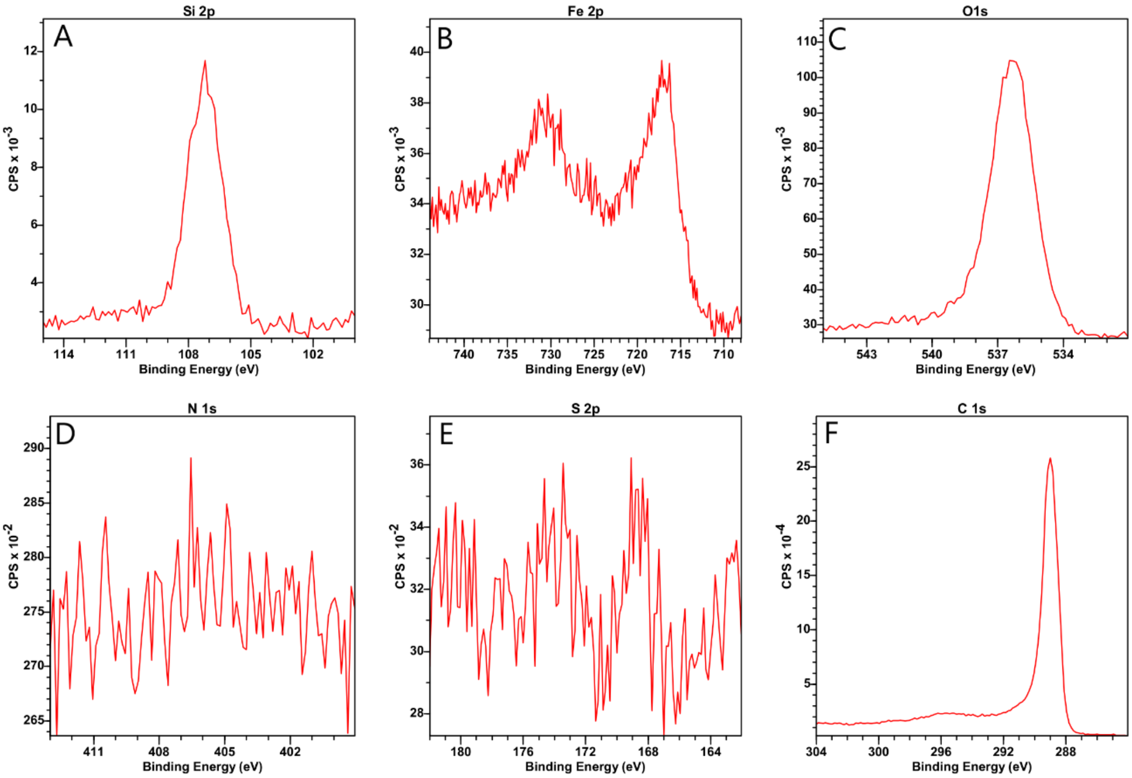

Figure S6. Narrow scan XPS spectra of graphite treated with **plasma O_2**, 10 min and immersed in **water**. A) Si 2p, B) Fe 2p, C) O 1s, D) N 1s, E) S 2p, F) C 1s.

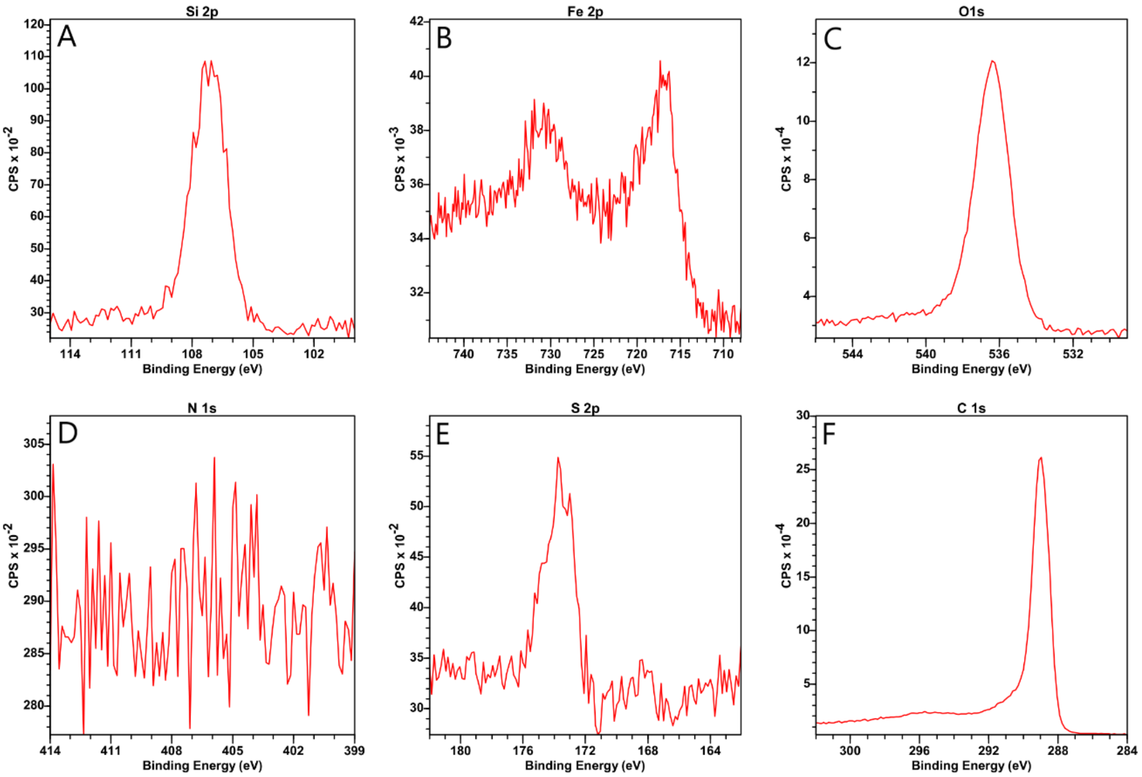

Figure S7. Narrow scan XPS spectra of graphite treated with **plasma CO_2**, 5 min – just after plasma treatment. A) Si 2p, B) Fe 2p, C) O 1s, D) N 1s, E) S 2p, F) C 1s.

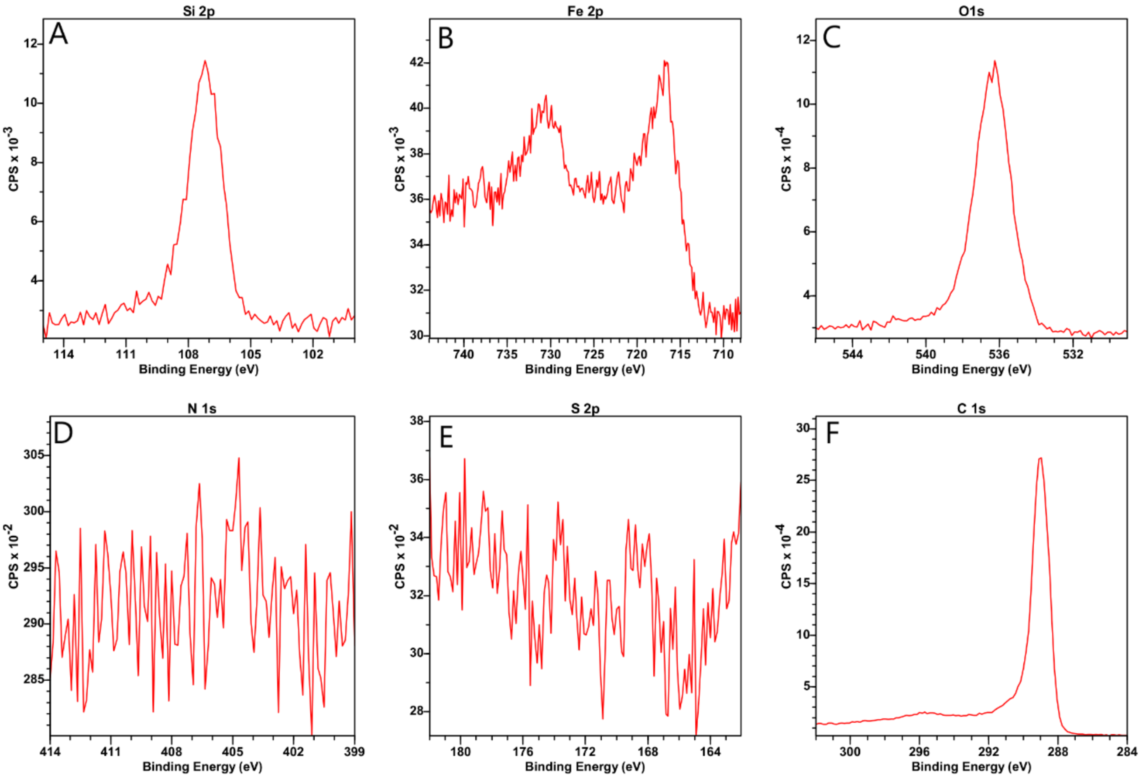

Figure S8. Narrow scan XPS spectra of graphite treated with **plasma CO_2**, 10 min and immersed in water. A) Si 2p, B) Fe 2p, C) O 1s, D) N 1s, E) S 2p, F) C 1s.

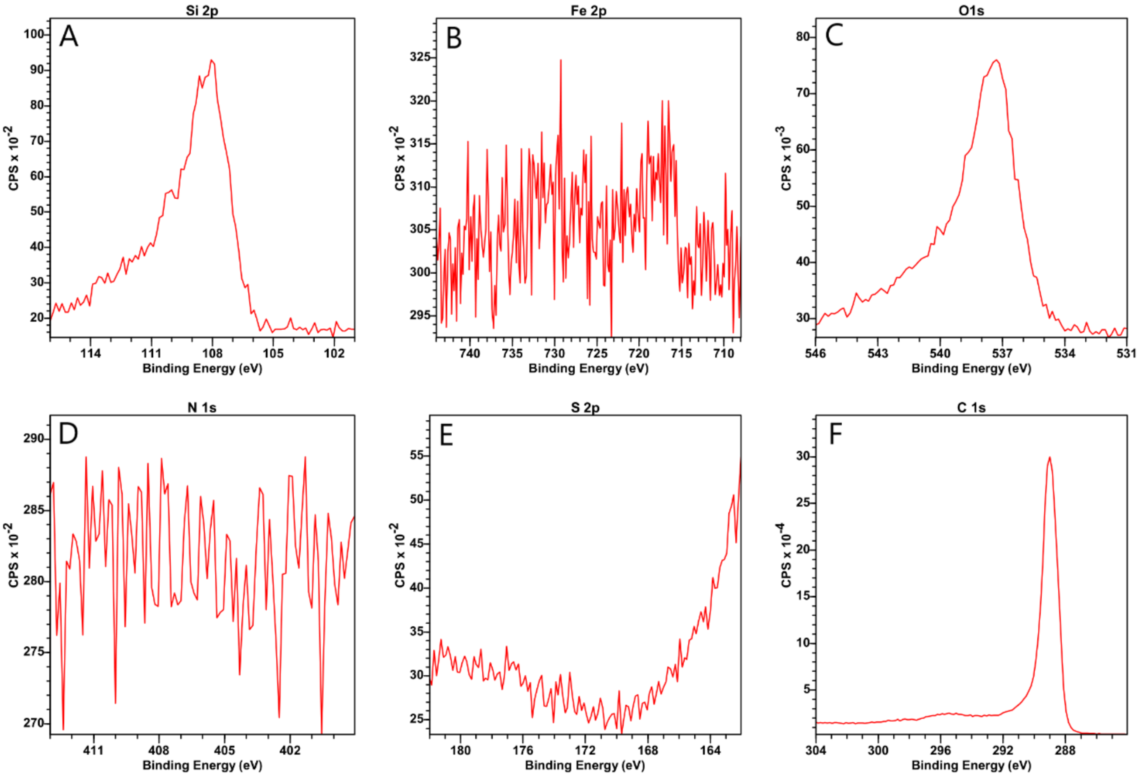

Figure S9. Narrow scan XPS spectra of graphite treated with **nitric acid.** A) Si 2p, B) Fe 2p, C) O 1s, D) N 1s, E) S 2p, F) C 1s.

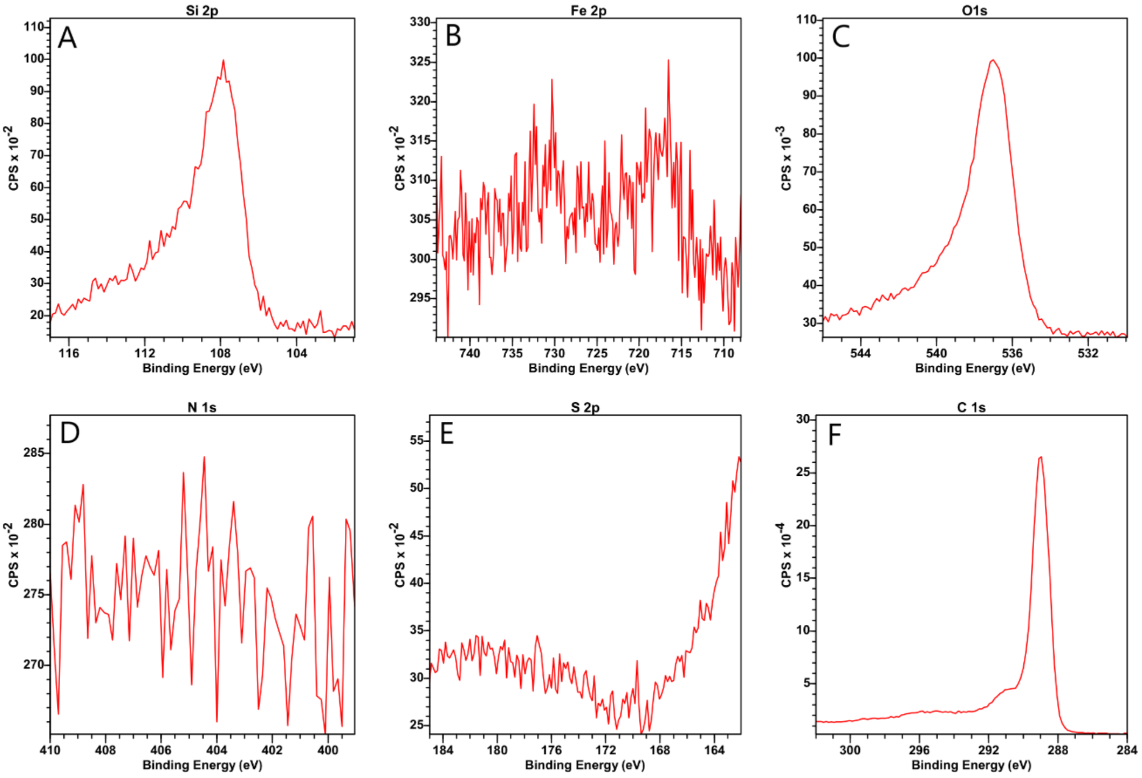

Figure S10. Narrow scan XPS spectra of graphite treated with **nitric acid** and **plasma O_2** – just after plasma treatment. A) Si 2p, B) Fe 2p, C) O 1s, D) N 1s, E) S 2p, F) C 1s.

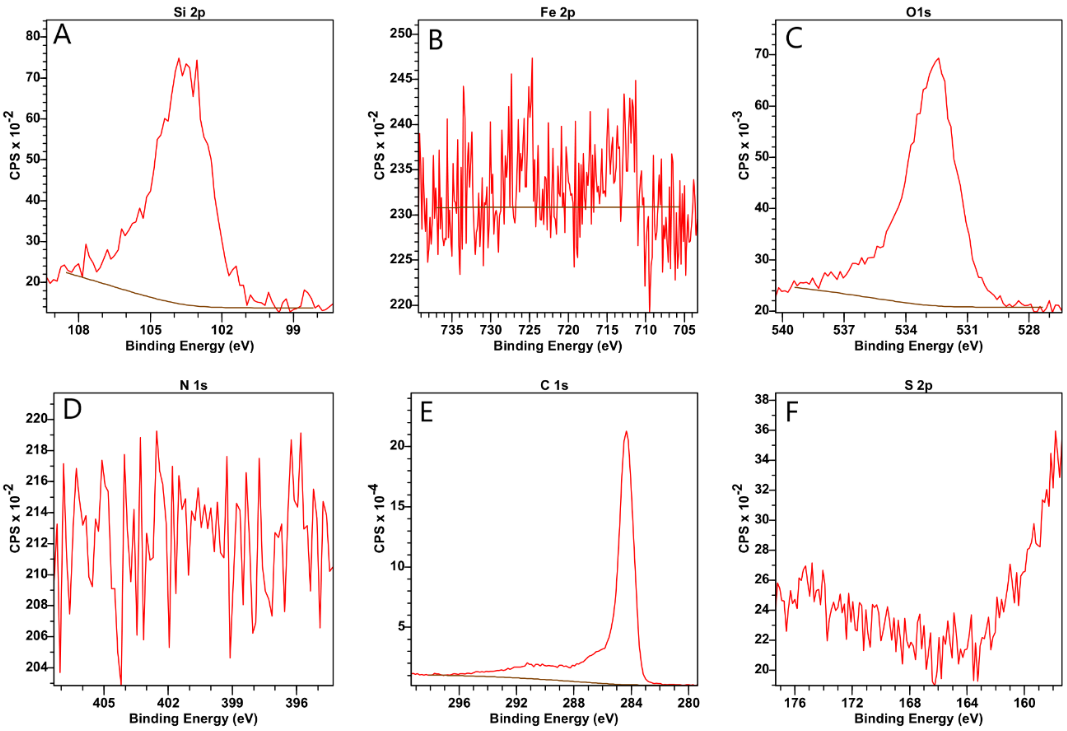

Figure S11. Narrow scan XPS spectra of graphite treated with **nitric acid** and **plasma O_2** and immersed in **water.** A) Si 2p, B) Fe 2p, C) O 1s, D) N 1s, E) C 1s, F) S 2p.

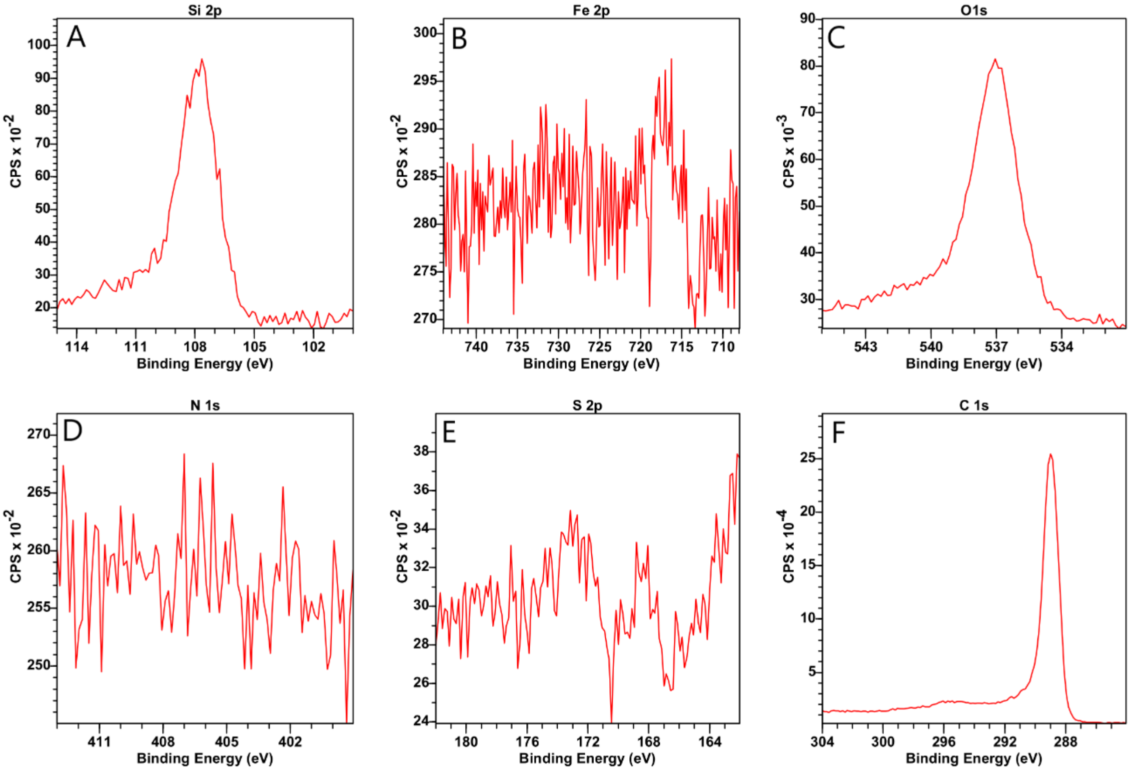

Figure S12. Narrow scan XPS spectra of graphite treated with **sulfuric acid.** A) Si 2p, B) Fe 2p, C) O 1s, D) N 1s, E) S 2p, F) C 1s.

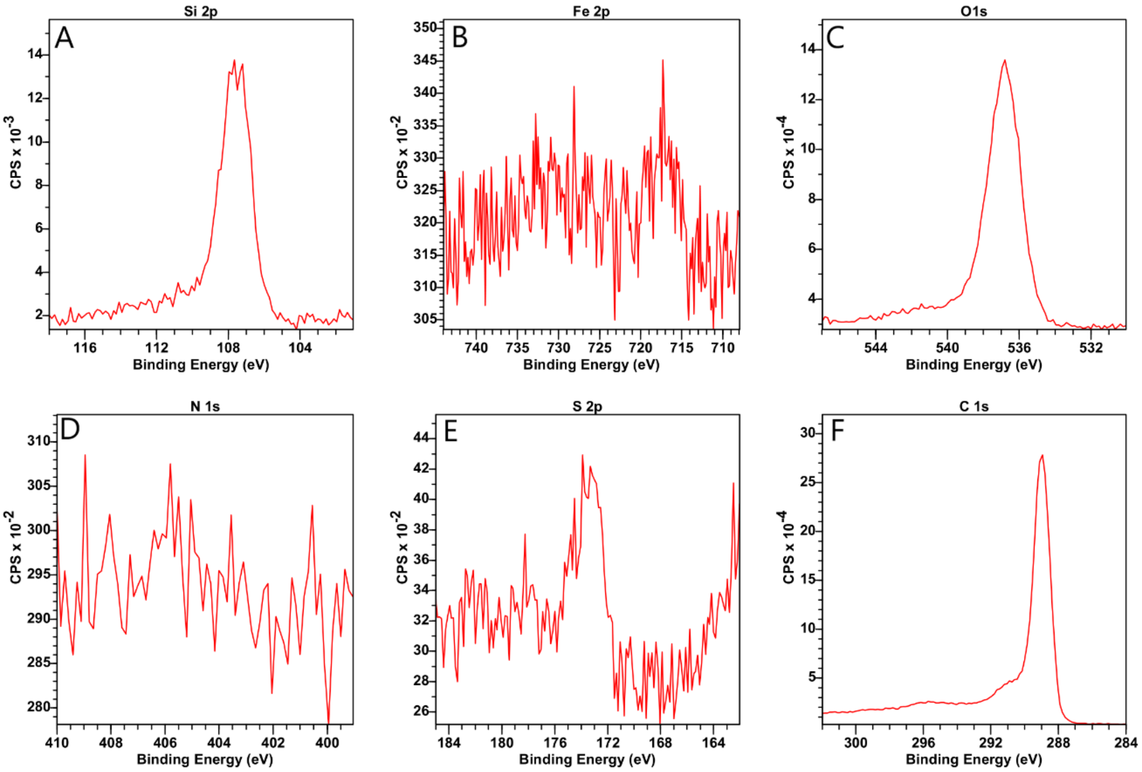

Figure S13. Narrow scan XPS spectra of graphite treated with **sulfuric acid** and **plasma O_2** – just after plasma treatment. A) Si 2p, B) Fe 2p, C) O 1s, D) N 1s, E), S 2p F) C 1s.

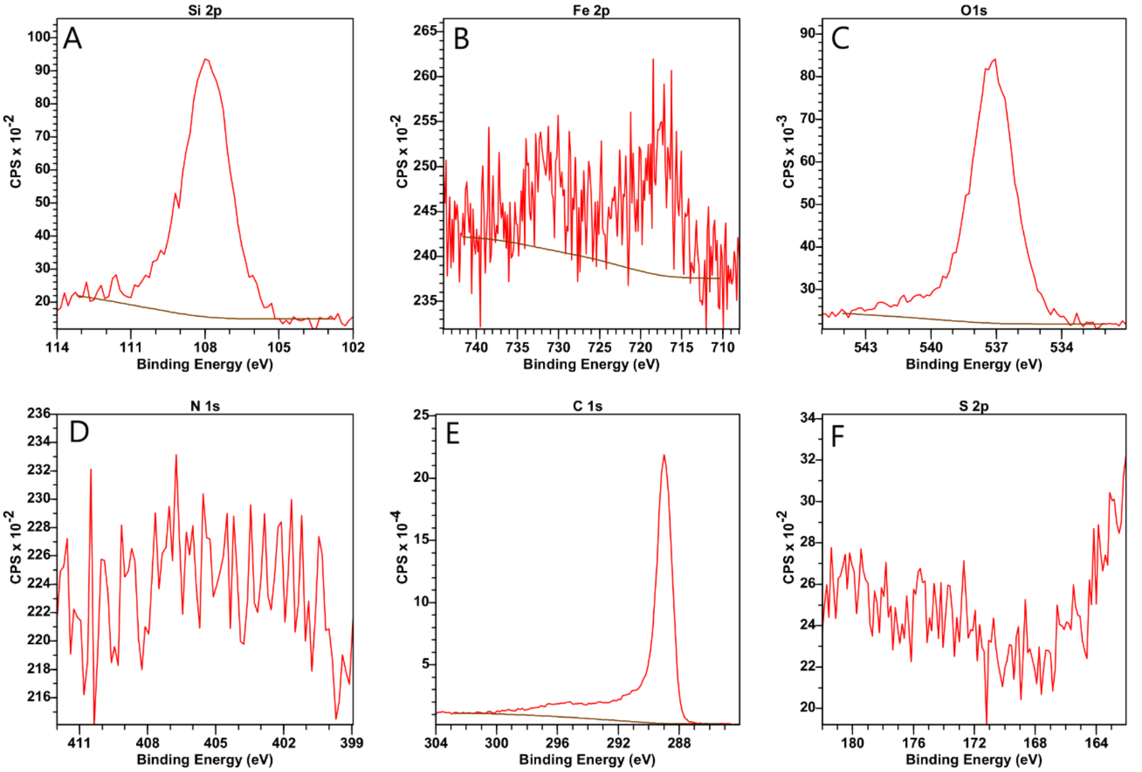

Figure S14. Narrow scan XPS spectra of graphite treated with **sulfuric acid** and **plasma O_2** and immersed in **water**. A) Si 2p, B) Fe 2p, C) O 1s, D) N 1s, E) C 1s, F) S 2p.
